# Effective fat-suppression for late gadolinium enhancement combined with a sequential acquisition order

**DOI:** 10.1186/1532-429X-11-S1-P75

**Published:** 2009-01-28

**Authors:** Dana C Peters, Basem Dokhan, Reza Nezafat, Yuchi Han, Evan Appelbaum, Warren J Manning

**Affiliations:** 1 Beth Israel Deaconess Medical Center and Harvard Medical School, Boston, MA USA; 2 ETH, Biomedical Engineering, Zurich, Switzerland

**Keywords:** Late Gadolinium Enhancement, Mitral Valve Prolapse, Centric Order, Adiabatic Pulse, Acquisition Order

## Introduction

Fat suppression is valuable for late gadolinium enhancement (LGE) imaging, for situations in which an enhanced structure is adjacent to fat (e.g. myocardium at the apex of the left ventricle, vessel walls, and the thin atrial walls), where fat might be mistaken for fibrosis. However, the acquisition order of the segmented LGE sequence is also critical, and a sequential acquisition order is superior to a centric acquisition order when visualizing small areas of scar, in which the "edge-enhancement" (i.e. the stronger weighting of the edges of k-space due to signal regrowth during the acquisition window) effect is not desired. A fat-suppressed LGE sequence using a sequential acquisition order may be optimal, but is not feasible using the typical fat-selective (spectrally-selective) RF pulse with a flip angle from 90° to 180° preceding the sequential acquisition. To address this, we developed a "fat restore" method for fat-suppressed LGE.

## Methods

**Figure 1:**
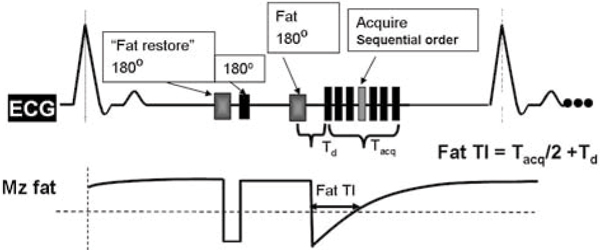
**The LGE sequence is modified so that a "fat restore" pulse precedes the first inversion pulse, and fat is not inverted**. This permits the 2^nd^ fat-selective inversion pulse to be effective in providing fat-suppression for a sequential acquisition order.

Without a "fat restore" pulse, the fat experiences two 180°s. The 1^st^ 180° (the non-selective inversion always used in LGE) results in greatly reduced M_zfat_ at the time of the 2^nd^ fat-selective180°. The result is that fat regrows very quickly after the 2^nd^ fat-selective180° and fat-suppression is not achievable for a sequentially ordered acquisition: fat crosses the null point too early, requiring a very short fat TI (the time between the fat suppression pulse and the acquisition of the center of k-space, defined in Figure 1). However, the minimum fat TI is ~Tacq/2 for a sequential acquisition order. The "fat-restore" method uses an additional fat-selective 180° before the standard non-selective 180° used for LGE. The purpose of the "fat restore" pulse is to tip down the fat so that subsequent 180° tips it back up to full magnetization. This will permit the later fat-selective 180° to be effective (see schematic in Figure 1). Phantoms including fat were imaged with and without the fat-restore pulse. Finally, the method was used in patient LGE studies. Scan parameters for phantoms and human studies were: 60 bpm, TR/TR/flip = 4.3–5 ms/1.6–2.3 ms/20°, 32–40 views per segment (Tacq ~180 ms), sequential order, 1 RR per inversion, TI set to null myocardium at the center of k-space (250 ms for phantoms). The fat TI was set to 100 ms using the fat-restore pulse. Without the fat-restore pulse, the minimum achievable fat TI (~100 ms also) was used. For patients, 0.2 mmol/kg Gd-DTPA was injected, and scanning was performed 15–2 minutes post injection. All spectrally selective 180° pulses were adiabatic pulses. The spatial resolution was 2 × 2 × 8 mm for 2D LGE.

## Results

**Figure 2:**
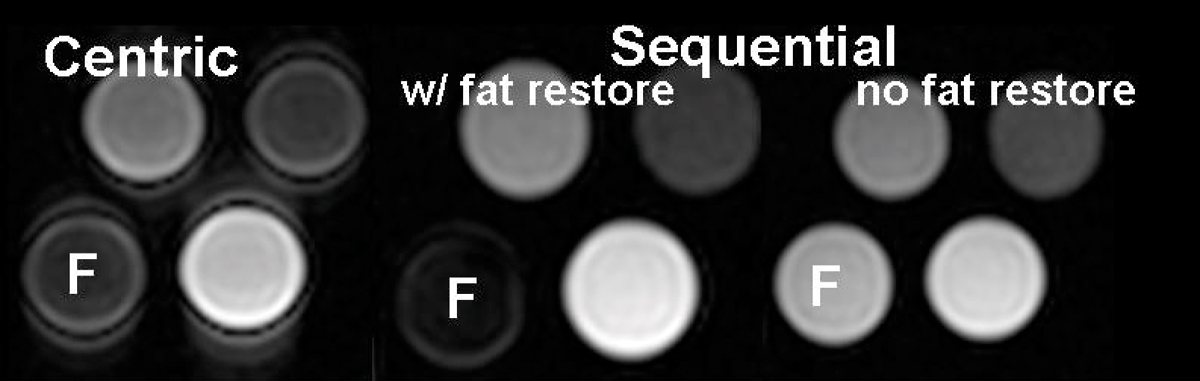
**Phantom comparison of the fat suppression using standard method with centric order, and the sequential order with and without the fat restore pulse**. Equivalent fat-suppression is provided by the fat restore technique. Edge enhancement is less using a sequential order. F = fat.

**Figure 3:**
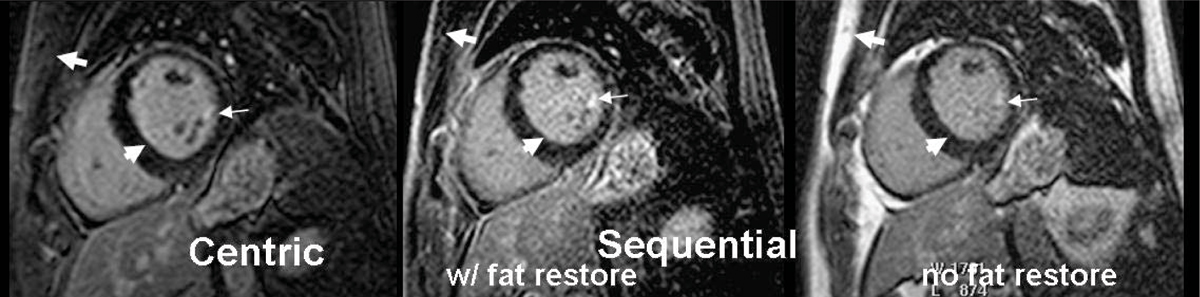
**2D LGE comparing matched slices using centric, and sequential orders with and without the fat-restore pulse**. The fat-restore pulse is needed for fat-suppression (thick arrows). Subtle edge-effects can be seen in the image with a centric acquisition (arrow heads). An area of enhancement at the tip of a papillary muscle (confirmed in other views) is also visualized in each image (thin arrows).

In phantom studies, the optimal fat TI (Figure 1) was ~120 ms and the ratio of SNR_fat w/fatsat_ to SNR_fat w/out fatsat_ was 8%, using the fat restore pulse; without the additional pulse the ratio was ~100%. Figure 2 shows that the fat restore pulse with a sequential acquisition order provided similar fat-suppression as the standard fat-saturation using centric order with reduced edge enhancement. Furthermore, without a fat-restore pulse, fat-suppression was not good. Figure 3 compares a fat-suppressed 2D LGE scan with centric order and sequential order, with and without the fat restore pulse, in a patient with mitral valve prolapse. The subcutaneous fat is well suppressed even for the sequential order (thick arrows) using the fat restore pulse. A region of confirmed enhancement can be seen in all images (thin arrows), but a edge-enhancement effect is seen only in the image with centric acquisition order (arrow-heads).

## Discussion and conclusion

We have demonstrated that the fat-restore pulse improves fat-suppression for sequentially acquired LGE scans. Studies with 3D LGE will likely improve with the linear acquisition order, improving the confidence for detecting small regions of scar.

